# SARS-CoV-2 Transmission Control Measures in the Emergency Department: The Role of Rapid Antigenic Testing in Asymptomatic Subjects

**DOI:** 10.3390/healthcare10050790

**Published:** 2022-04-23

**Authors:** Marina Borro, Gerardo Salerno, Andrea Montori, Andrea Petrucca, Paolo Anibaldi, Adriano Marcolongo, Rita Bonfini, Maurizio Simmaco, Iolanda Santino

**Affiliations:** 1 Department of Neurosciences, Mental Health and Sensory Organs (NESMOS), Sapienza University, Via di Grottarossa 1035/1039, 00189 Rome, Italy; maurizio.simmaco@uniroma1.it (M.S.); iolanda.santino@uniroma1.it (I.S.); 2 Laboratory of Clinical Biochemistry, Sant’Andrea University Hospital, Via di Grottarossa 1035, 00189 Rome, Italy; andrea_petrucca@yahoo.com; 3 Microbiology Unit, Sant’Andrea University Hospital, Via di Grottarossa 1035, 00189 Rome, Italy; andrea.montori@uniroma1.it; 4 Medical Direction, Sant’Andrea University Hospital, Via di Grottarossa 1035, 00189 Rome, Italy; paolo.anibaldi@ospedalesantandrea.it; 5 General Direction, Sant’Andrea University Hospital, Via di Grottarossa 1035, 00189 Rome, Italy; amarcolongo@ospedalesantandrea.it; 6 Emergency Department Unit, Sant’Andrea University Hospital, Via di Grottarossa 1035, 00189 Rome, Italy; rita.bonfini@ospedalesantandrea.it

**Keywords:** rapid SARS-CoV-2 antigen testing, emergency department, asymptomatic COVID-19, COVID-19 transmission control

## Abstract

Limiting transmission of SARS-CoV-2 from asymptomatic people assumes the paramount importance of keeping fragile subjects protected. We evaluated the utility of rapid SARS-CoV-2 antigen testing in asymptomatic subjects attending emergency departments in non-COVID-19 areas, using a single nasopharyngeal swab specimen collected in universal transport medium to perform both rapid antigen testing and rRT-PCR (used as reference standard) in a cohort of 899 patients. In the overall sample, the rapid antigen test had 43.9% sensitivity, 100% specificity, 100% positive predictive value, 93.6% negative predictive value. Considering subjects with rRT-PCR cycle threshold ≤30, the test had 80.4% sensitivity, 100% specificity, 100% positive predictive value, 98.8% negative predictive value. Considering subjects with rRT-PCR cycle threshold ≤25, the test had 94.7% sensitivity, 100% specificity, 100% positive predictive value and 99.7% negative predictive value. Despite low sensitivity, routine application of rapid antigen testing in the emergency department can lead to isolation in less than 30 min of about a half of asymptomatic COVID-19 subjects assigned to non-COVID-19 areas by clinical triage. The rapid test correctly identified 94.7% of asymptomatic patients with cycle threshold ≤ 25 that are supposed to be more infective; thus, it could be a useful measure to contain viral transmission in non-COVID-19 areas.

## 1. Introduction

The SARS-CoV-2 Omicron variant (B.1.1.529), originally identified in South Africa [1], has been associated with decreased severity [2,3] but higher transmission rates [4]. The landscape of the Omicron mutations within the antigenic spike protein enhances the escape from immune response induced by vaccination [5]; still, vaccines have been proven to maintain sustained protection against symptomatic disease and hospitalization [6,7]. In countries with broad vaccination coverage, the diffusion of the Omicron variant increased the prevalence of undiagnosed infections in asymptomatic and pauci-symptomatic subjects, contributing to viral spread. Thus, early detection of SARS-CoV-2 infection remains a main strategy for pandemic containment. 

The “gold standard” for COVID-19 diagnostics is the detection of SARS-CoV-2 nucleic acid by real-time reverse transcription PCR (rRT-PCR) from nasopharyngeal (NP) swab material. rRT-PCR is a lab-based assay with high sensitivity but requires skilled personnel, has relevant costs and relatively long turnaround time (TAT), that is, several hours [8]. A plethora of other diagnostic methods, based on detection of viral nucleic acid, viral antigens or host antibodies have been developed [8,9,10,11], attempting to reduce the diagnostic TAT. The high pressure on diagnostic centers posed by increasing demand for SARS-CoV-2 testing stimulated the development of several point-of-care testing (POCT) devices, mainly based on rapid detection of viral antigens (AgPOCT) in nasopharyngeal swabs [12,13,14]. Despite the technical issues limiting their accuracy, such devices are cheap, rapid (TAT of less than 30 min) and can be performed everywhere by non-skilled personnel [12]. SARS-CoV-2 POCT is widely used in a variety of settings including large scale screening [15,16,17], surveillance [18,19] and prevention [20,21]. 

In hospital environments, SARS-CoV-2 AgPOCT is employed with caution and its appropriateness, particularly in the emergency departments (EDs), is debated [22]. From the beginning of the COVID-19 pandemic the EDs, as frontline hospital divisions providing urgent medical assistance to patients who attend without prior appointment, faced an unparalleled challenge due to the conflicting priorities of both rapid patient treatment and patient protection from SARS-CoV-2 transmission. Organizational models providing different areas to manage patient ED attendance according to clinical suspicion of COVID-19, followed by patient sorting according to SARS-CoV-2 molecular diagnosis, have been promptly set up [23,24,25,26]. In such a setting, the incorrect sorting of SARS-CoV-2 asymptomatic subjects is an additional concern due to the long TAT (about 3 h in an emergency setting) for rRT-PCR, yet the sole application of AgPOCT in COVID-19 asymptomatic patients is not acceptable due to the low accuracy. A doubled test strategy using both AgPOCT and rRT-PCR in the ED could be a compromise: considering that AgPOCT testing usually has high sensitivity for subjects with high viral load [27], AgPOCT devices could rapidly identify at least this fraction of ED patients, allowing immediate allocation to reserved clinical areas and decreasing the volume of patients awaiting the rRT-PCR results. 

This study aimed to evaluate the suitability of such an approach in the ED of the Sant’Andrea Hospital of Rome, Italy, using the Green Spring “SARS-CoV-2 Antigen Rapid Test Kit (Colloidal Gold)” (Shenzhen Lvshiyuan Biotechnology Co., Ltd., Shenzhen, China) coupled with rRT-PCR. To reduce the discomfort associated with doubled NP swab collection, rRT-PCR and rapid testing were performed on the same specimen.

## 2. Materials and Methods

### 2.1. SARS-CoV-2 rRT-PCR Assay

Nasopharyngeal swabs collected in universal transport medium (UTM, Copan Diagnostics Inc., Murrieta, CA, USA) were analyzed using the automated platform for RNA extraction and amplification Versant kPCR (Siemens Healthineers AG, Erlangen, Germany) using the FTD SARS-CoV-2 Assay (Fast Track Diagnostics, Luxembourg), targeting the viral N gene and the ORF1ab region. All procedures were performed according to the manufacturer’s instructions. Samples showing amplification at a cycle threshold (Ct) ≥40 were considered as negative result. 

### 2.2. SARS-CoV-2 Antigen Rapid Test

The Green Spring “SARS-CoV-2 Antigen Rapid Test Kit (Colloidal Gold)” (Shenzhen Lvshiyuan Biotechnology Co., Ltd., Shenzhen, China) is a lateral flow test intended for SARS-CoV-2 detection in nasal swab specimens collected and plunged into a sample buffer tube included in the kit. The manufacturer declares 90% sensitivity and 99% specificity using rRT-PCR as the standard reference. As detailed below, the rapid test was performed (i) according to the manufacturer’s instructions (standard protocol), or (ii) using three drops of the NP material collected in UTM for rRT-PCR (UTM-modified protocol).

In the preliminary phase of the study, we compared the performance of the standard and UTM-modified protocol using rRT-PCR as the reference standard. Hence, from December 2021 to January 2022, we tested 300 subjects (validation cohort) who underwent, at the same time, paired NP swab collection: the first swab was collected in the Green Spring sample buffer, the second in UTM. The latter was used for both rapid antigen testing and for rRT-PCR. Then, results of the paired Green Spring rapid assays (standard and UTM-modified protocol) were compared with results from rRT-PCR, and their specificity and sensitivity were calculated, first in the overall population then including only rRT-PCR positive samples with Ct ≤ 30. 

### 2.3. Evaluation of Doubled Testing of UTM-Collected NP Swabs at the Emergency Department

A prospective evaluation of the doubled testing strategy was performed on samples collected between 23 January and 3 March 2022. Patients admitted to the ED underwent immediate NP swab UTM-collection, triage and allocation to the “COVID-19” area or to the “non-COVID-19” area according to the presence/absence of symptomatology suggestive for SARS-CoV-2 infection. NP swab specimens were delivered to the microbiology laboratory, where rapid antigen testing and rRT-PCR were set-up as an urgent priority. The study included 899 subjects (476 males, 423 females, mean age 60.5 ± 22.97). Using the rRT-PCR results as the reference standard, specificity and sensitivity of the rapid antigen test were calculated, first in the overall population then stratifying according to an rRT-PCR Ct ≤ 30 or an rRT-PCR Ct ≤ 25.

## 3. Results

### 3.1. Performance of the UTM-Modified Protocol for Rapid Antigen Testing

Among the validation cohort, the rRT-PCR assay detected 99 positive subjects (33%) with a mean Ct of 24.9 ± 6.45 (range 13.5–39.7); 79 of them were AgPOCT positive using the standard protocol, and 70 of them were AgPOCT positive using the UTM-modified protocol. Thus, the sensitivity of the rapid antigen test performed on UTM-collected NP swabs is reduced compared to the sensitivity obtained using the standard protocol (70.7% vs. 79.8%, Table 1), whereas the specificity of both the standard and UTM-modified protocol is 100%. 

Considering only positive subjects with an rRT-PCR Ct ≤ 30, the AgPOCT sensitivity was 96.1% and 85.7% using the standard or UTM-modified protocol, respectively, whereas the specificity was 100% for both methods. Importantly, the positive predictive value (PPV) for both AgPOCT protocols was 100%; that is, all subjects with positive antigen tests are true positive subjects (Table 1). 

### 3.2. Performance of Doubled Testing in Subjects Attending the Emergency Department

Out of 899 subjects (asymptomatic for COVID-19) included in the study, 98 (10.9%) received a diagnosis of SARS-CoV-2 infection by rRT-PCR, with a mean Ct value of 28.7 ± 7.84 (range 14–39), which was significantly higher than the mean Ct value in the validation cohort (*t*-test, *p* < 0.0001). Out of 98 rRT-PCR positive subjects, 43 (43.9%) were also correctly identified by the rapid antigen test performed on the same UTM-collected specimen. Thus, the overall sensitivity of the rapid antigen test in the ED cohort was 43.9%, but it increased drastically to 80.4% and 94.7% considering only subjects with rRT-PCR Ct ≤ 30 or Ct ≤ 25, respectively (Table 2). The specificity of the rapid test, as well as the PPV, was 100% in all the mentioned groups.

## 4. Discussion

Rapid identification and isolation of SARS-CoV-2 infected subjects remain a main measure to contain spreading of COVID-19. In Italy, the Omicron variant has presently 99% prevalence and is highly diffusive. The broad vaccination coverage is associated with decreased disease severity, with lower hospitalization rate and fewer deaths. On the other hand, the higher rate of asymptomatic or pauci-symptomatic infections [28] sustains viral diffusion and exposes fragile subjects to increased risk. Despite adoption of protection measures such as wearing masks, physical distancing and hand hygiene, patient attendance to the ED represents a critical milieu for SARS-CoV-2 transmission. In our ED, at the patient’s arrival, the personnel proceed to NP swab collection and quick triage for COVID-19 symptoms followed by partitioning in the COVID-19 area or non-COVID-19 area according to the clinical suspicion while the confirmatory rRT-PCR assay is pending from the laboratory. This means that infected but asymptomatic subjects can be allocated in the non-COVID-19 area, increasing the risk of viral diffusion. Clearly, shortening the time to COVID-19 diagnosis would be crucial to better management of the isolation of asymptomatic patients and to ensure a safe environment in a high-risk setting such as the ED, either for healthcare workers or vulnerable subjects. Unfortunately, rapid diagnostic tools such as AgPOCT devices are not sufficiently accurate to represent safe alternative to the gold standard rRT-PCR. Here, we tested the utility of a combinatorial approach to SARS-CoV-2 diagnosis in the ED, that is performing a doubled test, rapid antigenic and rRT-PCR, starting from the same NP specimens collected at the patient’s arrival. Although the use of UTM-collected specimens is usually not recommended by AgPOCT manufacturers, we considered that execution of two consecutive swabs would cause a non-negligible discomfort for patients, particularly for ED attending patients, and that it could increase the risk of improper specimen collection, affecting test results. Thus, we performed preliminary evaluation of the loss of accuracy of the AgPOCT performed in UTM swabs, comparing the results obtained in paired samples analyzed using the standard or an UTM-modified protocol. As expected, the AgPOCT overall sensitivity lowered from 79.8% to 70.7%, but considering subjects with higher viral load (Ct ≤ 30), the modified protocol achieved 85.7% sensitivity. Both specificity and PPV remained unchanged with a 100% value. This was a key observation, implying that all AgPOCT positive subjects were true positive and could be safely and rapidly assigned to COVID-19 areas. Thus, we proceeded to evaluate the utility of doubled testing strategy in the ED, studying a series of 899 patients with no symptoms of COVID-19 and thus preliminarily allocated in the non-COVID-19 area. The overall sensitivity of rapid antigen testing in this group of patients was lower (43.9%) compared to that obtained in the validation cohort (70.7%). This discrepancy can be related to the lower mean Ct values detected in the ED cohort compared to the validation cohort (28.7 ± 7.8 vs. 24.9 ± 6.4), as the AgPOCT sensitivity increases with the viral load expressed in Ct (Table 1 and Table 2). However, a limitation of this study is that the validation cohort was enrolled in a period when the Delta SARS-CoV-2 variant was still prevalent in Italy, while the ED cohort was mainly enrolled after the rapid shift of prevalence from Delta to Omicron variant. Thus, an effect of SARS-CoV-2 variants on the AgPOCT sensitivity cannot be ruled out. Nevertheless, translating numbers into real-world practice, a 43.9% sensitivity in the ED cohort means that 43 out of 98 infected but asymptomatic patients would have been isolated from non-infected subjects within 30 min from their arrival, approximatively halving the risk of transmission in an ED area supposed to be COVID-19 free. Moreover, 36 out of the 43 infected subjects identified by rapid antigen testing had high viral load (RT-PCR Ct ≤ 25) and thus are supposed to be more infective. They represented the 94.7% of all patients with an rRT-PCR Ct ≤ 25 (Table 2). Other studies evaluated the use of antigen rapid testing in the ED. In a pediatric ED, Ollier et al. [29] found that antigen testing using the BioSpeedia immunochromatographic assay had acceptable sensitivity (up to 100% in strongly positive patients) and specificity (near 100%) and thus support its use to improve management of symptomatic pediatric patients. More recently, third generation AgPOCTs based on microfluidic assays were evaluated in ED settings with discordant results [30,31,32]. 

Clearly, the use of AgPOCT in the ED, with the aim to reduce the SARS-CoV-2 diagnostic TAT, should be planned and carried out following stringent but dynamic rules, according to the rapidly evolving scenario of the COVID-19 pandemic. First, several AgPOCTs have been developed [33,34] and their actual level of accuracy should be carefully reassessed when novel SARS-CoV-2 variants emerge and become prevalent. Second, the pressure on ED mirrors the pandemic waves, so the risk/benefit ratio of AgPOCT testing can change drastically in mounting vs. declining periods. In symptomatic subjects during a pandemic peak, rapid AgPOCT testing added to clinical triage is probably enough to decide patient isolation without waiting for the rRT-PCR assay. Our data suggest that methodical employment of AgPOCT testing in the EDs may limit viral transmission by rapid identification and isolation of a significant fraction of infected but asymptomatic patients, with high viral load, who were incorrectly assigned to COVID-19 free areas.

## 5. Conclusions

Although rapid antigen tests are less sensitive than rRT-PCR, a strategic application in specific settings such as EDs may consistently contribute to limiting SARS-CoV-2 transmission among patients and operators and improve the overall diagnostic capacity.

## Figures and Tables

**Table 1:** Comparison of the diagnostic accuracy of standard and UTM-modified protocol for rapid antigen testing. Data are expressed as % values.

	TP	FP	TN	FN	PPV	NPV	Sensitivity	Specificity
**Standard protocol AgPOCT—Overall (N = 300)**	79	0	201	20	100	90.9	79.8	100
**UTM-modified protocol AgPOCT—Overall (N = 300)**	70	0	201	29	100	87.4	70.7	100
**Standard protocol AgPOCT—Ct ≤ 30 (N = 278)**	74	0	201	3	100	98.5	96.1	100
**UTM-modified protocol AgPOCT—Ct ≤ 30 (N = 278)**	66	0	21	11	100	94.8	85.7	100

**TP**: true positive; **FP**: false positive; **TN**: true negative; **FN**: false negative; **PPV**: positive predictive value; **NPV**: negative predictive value; **UTM**: universal transport medium.

**Table 2:** Evaluation of the diagnostic accuracy of the UTM-modified protocol for rapid antigen testing in the emergency department. Data are expressed as % values.

	TP	FP	TN	FN	PPV	NPV	Sensitivity	Specificity
**UTM-modified protocol AgPOCT—Overall (N = 899)**	43	0	801	55	100	93.6	43.9	100
**UTM-modified protocol AgPOCT—Ct ≤ 30 (N = 852)**	41	0	801	10	100	98.8	80.4	100
**UTM-modified protocol AgPOCT—Ct ≤ 25 (N = 839)**	36	0	801	2	100	99.7	94.7	100

**TP**: true positive; **FP**: false positive; **TN**: true negative; **FN**: false negative; **PPV**: positive predictive value; **NPV**: negative predictive value; **UTM**: universal transport medium.

## Data Availability

Not applicable.
